# Is There a Link between COVID-19 Mortality with Genus, Age, ABO Blood Group Type, and ACE2 Gene Polymorphism?

**DOI:** 10.18502/ijph.v49i8.3910

**Published:** 2020-08

**Authors:** Hamid CHEGNI, Nafiseh PAKRAVAN, Mojtaba SAADATI, Ali Dalir GHAFFARI, Hadi SHIRZAD, Zuhair M HASSAN

**Affiliations:** 1. Department of Immunology, School of Medical Sciences, Tarbiat Modares University, Tehran, Iran; 2. Department of Immunology, School of Medicine, Alborz University of Medical Sciences, Karaj, Iran; 3. Department of Biology, Imam Hossein University, Tehran, Iran; 4. Department of Parasitology, School of Medical Sciences, Tarbiat Modares University, Tehran, Iran; 5. Institute of Policy Research and Social Studies, Tehran, Iran

## Dear Editor-in-Chief

A group of pneumonia patients in Wuhan created an alarm worldwide by the end of December 2019. This emerging pneumonia soon became as a novel coronavirus (2019-nCoV or COVID-19) (1). More than 80% of individuals with COVID-19 have a “mild illness and will heal” whereas it is lethal in 2% of reported cases (2). The age and sex of individuals are two risk factors of COVID-19 susceptibility and its clinical outcome (3). It is therefore highly important to specify briskly the odds ratio for mortality adjusted to comorbidities that are highly prevalent globally, by age and sex, to prevent COVID-19 specific mortality (4).

We aimed to compute the relationship between the ABO blood type, age, sex, and ACE2 gene polymorphism with the susceptibility to COVID-19 in patients from Iran to test if the former can act as a biomarker for the latter.

Our study was performed on 94 random samples of dead cases infected with SARS-CoV-2. Of these, only 76 were identified by blood type. A recent survey of ABO blood group distribution of 80,982,137 normal people from Iran was used as comparison controls for patients with COVID-19 (https://en.wikipedia.org/wiki/Blood_type_distribution_by_country). Then, statistical analysis was performed using SPSS software version 15 (Chicago, IL, USA).

**Fig. 1:**
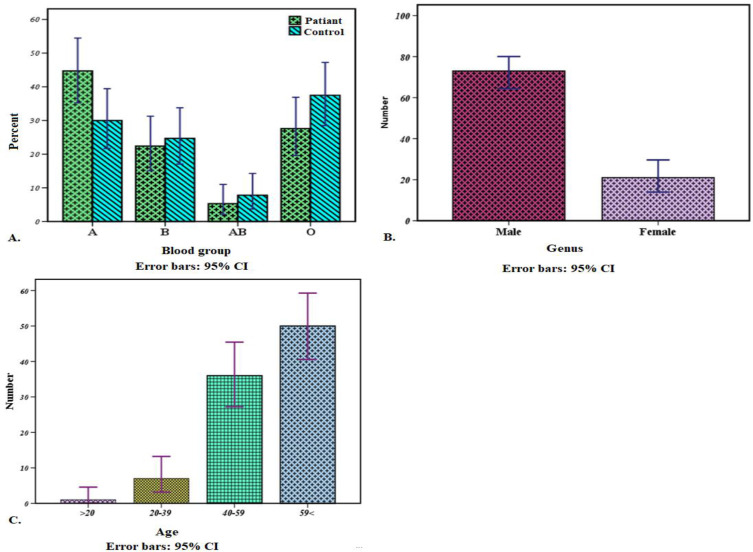
The chart of ABO blood group, genus, and age distribution in patients with COVID-19

The ABO blood group in 80,982,137 normal people and 76 dead patients with COVID-19 is showed in Table 1. People with type A phenotype were substantially more likely to become infected with 2019-nCoV (95% CI, *P*= 0.014, Fig. 1A). The O, AB and B blood groups seemed to have a lower risk of infection, although the associations did not reach statistical significance (Table 1).

**Table 1:** The relationship of COVID-19 with ABO blood group, age, and genus

***Variable***	***Blood Group***			
	***A***	***B***	***AB***	***O***
Controls (Iran)				
80,982,137	30%	24.7%	7.8%	37.5%
Death cases				
76	34 (44.7%)	17 (22.4%)	4 (5.3%)	21 (27.6%)
χ2	6.000	0.383	1.385	3.030
*P*	0.014	0.536	0.239	0.082
Genus				
Male	73 (77.7%)			
Female	21 (22.3%)			
χ2	28.766			
*P*	< 0.001			
Age (yr)				
<20	1(1.1%)			
20–39	7(7.4%)			
40–59	36 (38.3%)			
59<	50 (53.2%)			
Mann-Whitney Test $\frac{40-59}{59<}$ P value = 0.004				

We noticed that ABO blood groups exhibited various association risks for the infection with SARS-CoV-2 resulting in COVID-19. Our results similar to another one (3), in Wuhan, China showed blood group A was significantly associated with a higher risk of infection and blood group O was associated with a lower risk (3).

Moreover, we analyzed the people’s age and sex as two other risk factors among patients with COVID-19 where the COVID-19 mortality rate for men was four times more than that of women. The genus of dead patients was 73 men and 21 women. These results corresponded to a significantly increased risk of men in comparison with women for COVID-19 (Fig. 1B) (*P*< 0.001).

Also, current survey suggested that patients with ages of 50 < years are at greater risk relative to children who might be less likely to become infected or may show milder signs or even asymptomatic infection (*P*= 0.004). A dead case with the “age of 94 is also shown in Table 1 and Fig. 1C.

“Angiotensin-converting enzyme 2 (ACE2) is an enzyme attached to the outer surface (cell membranes) of cells in the lungs, arteries, heart, kidney, and intestines “(https://en.wikipedia.org/wiki/Angiotensin-converting_enzyme_2). ACE2 is known as SARS-CoV-2 receptor and balance of ACE-ACE2 axis and blood pressure are important in patients with COVID-19 and susceptibility to the disease. Association between genotype of ACE2-ACE axis, blood group A, and blood pressure has been previously reported (5, 6). Despite the genetic differences between Chinese and Iranian nations in ACE-ACE2 axis (7), people with blood group A in both nations are more susceptible to COVID-19 and risk of death.
